# Protein Kinase CK2 Inhibition Represents a Pharmacological Chance for the Treatment of Skin Diseases

**DOI:** 10.3390/ijms26115404

**Published:** 2025-06-04

**Authors:** Michele Scuruchi, Desirèe Speranza, Giuseppe Bruschetta, Federico Vaccaro, Mariarosaria Galeano, Giovanni Pallio, Mario Vaccaro, Francesco Borgia, Federica Li Pomi, Massimo Collino, Natasha Irrera

**Affiliations:** 1 Department of Clinical and Experimental Medicine, University of Messina, Via C. Valeria, 98125 Messina, Italy; mscuruchi@unime.it (M.S.); desiree.speranza@studenti.unime.it (D.S.); mario.vaccaro@unime.it (M.V.); francesco.borgia@unime.it (F.B.); federicalipomi@hotmail.it (F.L.P.); natasha.irrera@unime.it (N.I.); 2 Department of Veterinary Sciences, University of Messina, Viale G. Palatucci, 98168 Messina, Italy; giuseppe.bruschetta@unime.it; 3 Department of Biomedical and Dental Sciences and Morphological and Functional Imaging, University of Messina, Via C. Valeria, 98125 Messina, Italy; federico.vaccaro92@gmail.com (F.V.); mariarosaria.galeano@unime.it (M.G.); 4 Department of Neurosciences “Rita Levi Montalcini”, University of Torino, Via Verdi, 10124 Torino, Italy; massimo.collino@unito.it

**Keywords:** CK2 kinase, skin, inflammation, cancer

## Abstract

Protein kinase CK2 has emerged as a pivotal regulator of cellular processes involved in skin homeostasis, including cell proliferation, differentiation and inflammatory response regulation. In fact, CK2 activity dysregulation is implicated in the pathogenesis of different skin diseases, such as psoriasis, cancer and inflammatory dermatoses. CK2 overactivation fosters keratinocyte proliferation and pro-inflammatory cytokine production through the STAT3 and Akt pathways in psoriasis, thus contributing to epidermal hyperplasia and inflammation. In the realm of oncology, CK2 overexpression correlates with tumor progression, facilitating cell survival and metastasis in melanoma and non-melanoma skin cancers. Pharmacological inhibition of CK2 has demonstrated therapeutic potential, with CX-4945 (Silmitasertib) as the most studied adenosine triphosphate-competitive inhibitor (ATP-competitive inhibitor). Preclinical models reveal that CK2 inhibitors effectively mitigate pathological features of psoriasis, regulate keratinocyte differentiation, and suppress tumor growth in skin cancers. These inhibitors also potentiate the efficacy of conventional chemotherapeutics and exhibit anti-inflammatory effects in dermatological conditions. Future research will aim to enhance the specificity and delivery of CK2-targeting therapies, including topical formulations, to minimize systemic side effects. Combination therapies integrating CK2 inhibitors with other agents might offer synergistic benefits in managing skin diseases. This review underscores CK2’s critical role in skin and its therapeutic potential as a pharmacological target, advocating for innovative approaches to harness CK2 inhibition in dermatology.

## 1. Introduction

Skin diseases are among the most prevalent health problems globally, ranging from chronic inflammatory disorders, such as psoriasis, to life-threatening malignancies like melanoma. These conditions not only compromise the physical health of individuals but also have profound psychosocial impacts due to the reduction in quality of life [1,2]. Despite advances in dermatological therapies, a critical need still remains to discover new therapeutic targets to address current treatment limitations, which often exhibit variable efficacies since their use could be associated with side effects.

Protein Kinase 2 (CK2), a serine/threonine kinase, has emerged as a pivotal regulator of multiple cellular processes, including cell proliferation, survival, apoptosis, and inflammation [3,4]. Structurally, CK2 is a tetrameric enzyme composed of two catalytic (α or α′) and two regulatory (β) subunits; unlike many other kinases, CK2 is constitutively active and does not require upstream activation. This unique feature enables CK2 to be involved in a wide array of signaling pathways that are dysregulated in human diseases, including skin disorders [4]. CK2 overactivation has been observed in various dermatological conditions and contributes to pathophysiological processes, such as keratinocyte and fibroblast hyperproliferation, altered differentiation and pro-inflammatory cytokine production [5]. CK2 promotes epidermal hyperplasia and inflammation via the activation of signal transducer and activator of transcription 3 (STAT3) and Ak strain transforming/protein kinase B (Akt) pathways; its inhibition has been shown to manage symptoms in preclinical models [5]. Furthermore, CK2 involvement in cancer progression underscores its relevance in dermatological oncology, particularly in malignant conditions, such as cutaneous squamous cell carcinoma and melanoma [4].

In fact, recent studies have highlighted CK2 as a potential therapeutic target for the treatment of skin diseases. CK2 pharmacological inhibition has garnered significant attention as a promising strategy for treating CK2-associated diseases. In this context, preclinical and clinical studies have already demonstrated the efficacy of some CK2 inhibitors, such as CX-4945 (Silmitasertib), both in cancers and inflammatory disorders [6,7]. These inhibitors offer a dual advantage by targeting not only CK2 oncogenic functions but also modulating inflammatory responses, making CK2 particularly relevant for dermatological applications [5].

This review aims to explore CK2’s role in the pathogenesis of skin diseases and to evaluate the therapeutic potential associated with its inhibition. By integrating evidence from recent studies, we seek to provide a comprehensive understanding of how CK2 targeting might represent an innovative pharmacological approach to the management of dermatological conditions.

## 2. Protein Kinase CK2: Structure and Regulation of Different Cell Signaling

CK2 is a highly conserved serine/threonine protein kinase isolated from liver extracts as part of a mixture with CK1 kinase and first described in 1954 by Burnett and Kennedy; casein was used as an artificial substrate to identify its phosphorylating activity, which led to its original name, casein kinase 2. However, it was during the 1970s that CK2 was purified, and its subunit structure has been thoroughly characterized [8].

Structurally, CK2 is a tetramer consisting of two catalytic α or α′ isoforms and two regulatory β subunits. CK2α and CK2α′ are also active in the absence of CK2β; CK2α and CK2α′ exhibit catalytic activity even in the absence of CK2β. However, CK2β plays multiple essential roles, including CK2α isoform stabilization, substrate specificity regulation, and facilitating the assembly of functional complexes. These complexes show different forms, such as CK2α_2_β_2_, CK2αα′β_2_, or CK2α′_2_β_2_, depending on the combination of catalytic and regulatory subunits. Because of this ability, CK2 remains constitutively active even in its monomeric form [9,10]. Nevertheless, the predominant form in cells includes two catalytic α and/or α′ subunits, which associate with two non-catalytic β-subunits to form heterotetrameric complexes such as α_2_β_2_, αα′β_2_, or α′_2_β_2_ [11,12].

The human genome encodes four CK2 loci, comprising three codifying genes and a single pseudogene [13]. Among them, the *CSNK2A1* gene locus is located on chromosome 20p13 and encodes the catalytic α subunit (CK2α), the *CSNK2A2* gene locus encoding the catalytic α′ subunit (CK2α′) is located on chromosome 16p21, and the gene locus *CSNK2B* encoding the β subunits is located on chromosome 6p21 [13,14].

As mentioned, CK2 is constitutively active, and it is not directly influenced by signaling molecules, such as hormones and growth factors [15,16]. Consequently, CK2 does not participate in a linear transduction pathway. Instead, this kinase acts in a more interconnected manner, thus regulating multiple components across different signaling pathways in a lateral/horizontal fashion [15,16].

**Figure 1:**
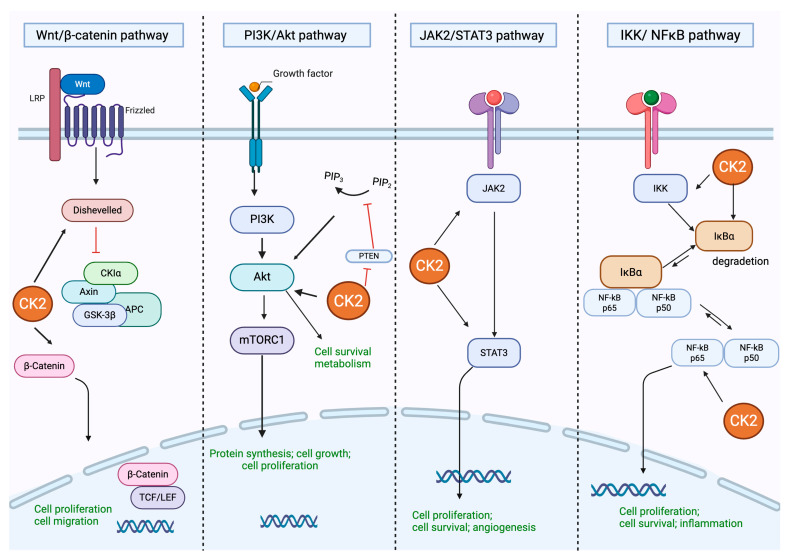
CK2 Modulation of Cellular Signaling Pathways: In the Wnt/β-catenin pathway, CK2 activates Dishevelled (Dvl), thereby inhibiting GSK3-mediated β-catenin degradation. CK2 also phosphorylates β-catenin, enhancing its stability, and targets TCF/LEF to promote the formation and transcriptional activity of the β-catenin/LEF complex. Within the PI3K/Akt pathway, CK2 directly enhances Akt activity while concurrently inhibiting PTEN, thereby attenuating its negative regulatory effects. In the JAK2/STAT3 pathway, CK2 activates both JAK2 and STAT3, while STAT3 reciprocally regulates CK2 expression. Additionally, in the IKK/NFκB pathway, CK2 promotes IκBα degradation, reducing its inhibitory effect, and activates both IKK and the p65 NFκB subunit, thereby enhancing NFκB signaling. Created in Biorender. Speranza. (2025) https://BioRender.com (accessed on 30 May 2025).

Given its constitutive activity and broad substrate specificity, CK2 plays a pivotal role in modulating multiple signaling pathways, including phosphoinositide 3-kinase/Ak strain transforming/protein kinase B (PI3K/AKT), Wingless/Integrated/β-catenin (Wnt/β-catenin), nuclear factor-kappa B (NF-κB), and the Janus kinase/ signal transducer; CK2 is an activator of transcription (JAK/STAT), p53, and Hedgehog, and transforms the growth factor-beta/suppressor of mothers against decapentaplegic (TGF-β/SMAD) pathways (Figure 1).

Through these pathways, CK2 regulates essential cellular processes such as proliferation, survival, apoptosis, stress response, and cell cycle regulation. Consequently, its deregulation has been associated with several diseases, making CK2 an attractive potential therapeutic target.

In fact, CK2 modulates the cell cycle and exerts anti-apoptotic effects by modulating the PI3K/AKT pathway at multiple levels. Specifically, it has been reported that CK2 phosphorylates AKT at Ser129 in the linker region. This modification facilitates Thr308 phosphorylation in the activation loop by phosphoinositide-dependent kinase-1 (PDK1), the upstream kinase responsible for canonical AKT regulation. Thr308 phosphorylation is followed by further phosphorylation at Ser473 in the hydrophobic domain, resulting in full activation of AKT. This cascade of events promotes cell survival and inhibits apoptosis [4,17,18,19]. Furthermore, CK2 sustains PI3K/AKT signaling by directly phosphorylating the phosphatase and tensin homolog (PTEN), thereby impairing its phosphatase activity and preventing the downregulation of the pathway. Notably, this regulation involves a functional interplay between CK2 and Glycogen Synthase Kinase-3 (GSK-3) [20]. Additionally, CK2 has been shown to enhance PI3K signaling pathway activation by phosphorylating and activating the transcriptional factor Ikaros, which in turn promotes the expression of genes involved in PI3K signaling [21]. Collectively, these findings indicate that CK2 regulates the PI3K/AKT pathways through two complementary mechanisms: post-translational modulation of AKT and PTEN and transcriptional regulation of pro-PI3K genes, forming a regulatory network that integrates both kinase activity and gene expression modulation.

Moreover, CK2 regulates NF-κB p65 transcriptional activity through multiple molecular mechanisms. One of the key mechanisms involves the phosphorylation of the NF-kappa-B inhibitor alpha (IKB-α), which promotes its proteasomal degradation and consequently facilitates NF-κB release. This event enhances NF-κB translocation from the cytoplasm to the nucleus, thus activating gene transcription [22]. In addition, CK2 directly phosphorylates the NF-κB p65 subunit at Ser529, with the consequent promotion of nuclear translocation and transcriptional activation [23]. Beyond these two phosphorylation events, CK2 also participates in a broader regulatory network that includes interactions with additional components of the NF-κB signaling machinery. For example, it modulates the activity of NF-κB essential modifier (NEMO)/IκB kinase-γ (IKKγ), a crucial adaptor protein that enables IκB phosphorylation and coordinates with other signaling pathways to fine-tune NF-κB activity at both post-translational and transcriptional levels [24,25,26].

CK2 plays a crucial role in the modulation of the JAK/STAT signaling cascade by directly interacting with JAK2 and influencing its activation status. Mechanistically, CK2 binds directly to JAK2 in a phosphorylation-independent manner and enhances its activity in response to cytokine stimulation, such as oncostatin M and interferon-gamma (IFN-γ) [27,28]. This interaction facilitates the phosphorylation of downstream effectors, including STATs and extracellular signal-regulated kinases (ERKs), thereby promoting the transcription of target genes, like the suppressor of cytokine signaling 3 (SOCS3) and the anti-apoptotic factor B-cell lymphoma-extra-large (Bcl-xL). Notably, CK2 directly phosphorylates JAK2, and this post-translational modification appears to be critical for the full activation of JAK2 and propagation of downstream signals. In fact, inhibition of CK2α and CK2β subunits markedly reduces JAK2 autophosphorylation and impairs STAT-dependent transcriptional responses [27,28].

CK2 contributes to the stabilization of β-catenin, a key transcriptional cofactor in the Wnt signaling pathway, which is involved in different cell mechanisms, including cell proliferation [29]. While GSK3 promotes β-catenin degradation through phosphorylation at the N-terminus, CK2 counteracts this action by phosphorylating β-catenin within its armadillo repeat domain, thereby protecting it from proteasomal degradation. Through this action, CK2 supports oncogenic Wnt signaling by enhancing β-catenin stability [29].

As a response to growth factors, quiescent cells can activate CK2 biosynthesis; in this setting, CK2 interacts with key intracellular molecules, thus modulating cell response to growth factors [30].

The mechanisms of cell proliferation and growth are dependent on cell cycle regulation: p53 protein is one of the most important cell cycle regulators. CK2 promotes p53 DNA binding activity following serine 386 phosphorylation on p53 C-terminal residues and enhances its ability to suppress cellular growth [31,32,33].

In the Hedgehog signaling pathway, CK2 acts as a positive regulator for the modulation of key components involved in signal propagation. CK2 enhances Hedgehog signaling by stabilizing a GPCR-like protein, smoothened (Smo), at the cell membrane level thanks to the phosphorylation of specific serine residues on the C-terminal cytoplasmic tail of Smo. Beyond Smo, CK2 also acts downstream by stabilizing full-length cubitus interruptus (Ci), a key transcriptional activator of Hedgehog target genes [34]. In addition, CK2 plays a pivotal role in promoting TGF-β-induced microtubule acetylation by directly activating α-TAT1, the primary α-tubulin acetyltransferase. CK2α binds to the C-terminal domain of α-TAT1 and phosphorylates Ser236, a modification essential for α-TAT1 catalytic activation. This CK2-dependent mechanism is required for proper TGF-β signaling through microtubule acetylation [35].

In summary, CK2 emerges as a master regulator of multiple signaling pathways that orchestrate essential cellular processes, including cell proliferation, apoptosis, and inflammation. Its constitutive activity, broad substrate range, and involvement in several molecular networks highlight its pivotal role not only in physiological regulation but also in the pathogenesis of different diseases, making it a compelling target for therapeutic intervention.

## 3. Role of CK2 in the Pathogenesis of Skin Diseases

Psoriasis is a chronic inflammatory skin disorder characterized by keratinocyte hyperproliferation and abnormal differentiation that contribute to scaly plaque formation [36]. Increased CK2 expression has been observed in psoriatic lesions, thus suggesting its involvement in the progression of the disease [5]. In fact, CK2 promotes keratinocyte proliferation and abnormal differentiation by modulating key signaling pathways, including STAT3 and Akt pathways [5]. Moreover, STAT3, together with NF-κB and AP-1 pathways, is involved in the production of pro-inflammatory cytokines, which play a key role in psoriasis, as well as in other inflammatory conditions, such as atopic dermatitis [37]. In this context, it has been shown that CK2 modulates the inflammatory response by regulating NF-κB and AP-1 and by reducing the consequent cytokine production [38]. The increased production of cytokines could also be a response to ultraviolet (UV) exposure, which may be involved in the initiation of skin carcinogenesis. UVB irradiation may stimulate the CK2-mediated IκB phosphorylation, which leads to NF-κB activation [22]. Therefore, CK2 inhibition could attenuate inflammatory responses in skin models, highlighting its potential in treating various inflammatory skin diseases.

For instance, some studies demonstrated that natural products, such as apigenin and ellagic acid, were able to inhibit CK2 activity/expression and, consequently, IκB phosphorylation induced by UVB [39].

**Figure 2:**
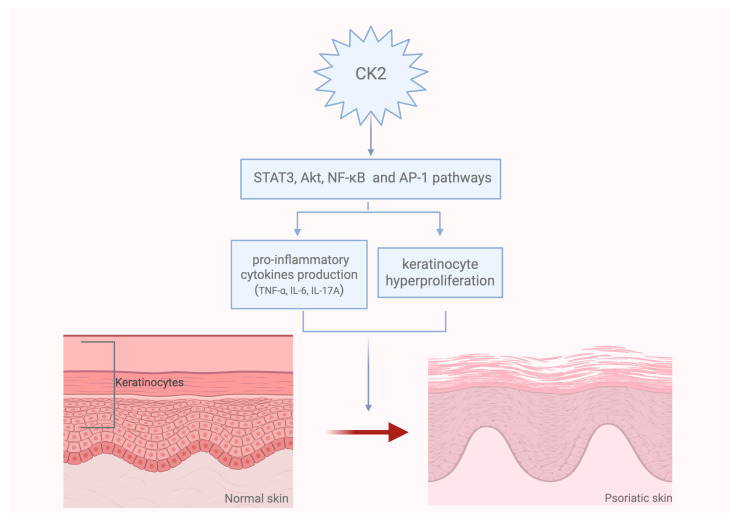
Schematic representation of CK2 involvement in the pathogenesis of psoriatic skin. CK2 activates several intracellular signaling pathways, including STAT3, Akt, NF-κB and AP-1. These pathways lead to increased production of pro-inflammatory cytokines, such as TNF-α, IL-6 and IL-17A, and hyperproliferation of keratinocytes, both of which are key to psoriasis pathogenesis. Created in Biorender. Speranza. (2025) https://BioRender.com (accessed on 27 April 2025).

Additionally, CK2 increased activity was observed in the skin lesions of psoriatic patients, so its inhibition could be effective in the management of psoriatic lesions. Preclinical studies indicated that the use of the CK2-specific inhibitor CX-4945 (Silmitasertib) was able to reduce the epidermal hyperplasia in a model of imiquimod (IMQ)-induced psoriasis through STAT3 and Akt modulation [40] as well as by c-Myc downregulation, whose activity is also involved in keratinocyte proliferation [41]. Upregulated CK2 expression is a contributing factor to the hyperproliferation of different cell lines, including keratinocytes. In fact, CK2 inhibitors could be useful in reducing cell proliferation through the regulation of involucrin, which is the precursor of the differentiation markers keratin 1 and 10. Together with this effect on abnormal keratinocyte proliferation, previous studies indicated that CX-4945 reduced the pro-inflammatory cytokine expression (IL-6, IL-17A, TNF-α) and, in particular, IL-17A, which plays a key role in psoriasis [5] (Figure 2). These pro-inflammatory cytokines are also involved in the development of other diseases that affect the skin, including systemic sclerosis (SSc). Systemic skin involvement is called diffuse cutaneous SSc (dcSSc), while sclerosis confined to the fingers, hands, and forearms is termed limited cutaneous SSc (lcSSc) [42]. Also, SSc is characterized by cell proliferation and uncontrolled fibroblast activation that induce excessive extracellular matrix deposition, thus disrupting tissue architecture. TGF-β is a key mediator of fibroblast activation in SSc and in other fibrotic diseases [43]. TGF-β promotes CK2α as well as CK2β expression, and TGF-β signaling inhibition may prevent CK2 upregulation in fibrotic skin [44]. As a feedback loop, CK2 activation may be essential for TGF-β pro-fibrotic effects on fibroblasts; therefore, CK2 inhibition may reduce extracellular matrix release mediated by TGF-β. CK2 inhibitors may prevent JAK2 phosphorylation, thus reducing STAT3 activation also in fibroblasts. The CK2 inhibitor TBB (4,5,6,7-tetrabromobenzotriazole) was effective in treating fibrosis by reducing collagen deposition and preventing fibroblast differentiation into myofibroblasts [45].

Although excessive collagen deposition may contribute to the onset of fibrotic conditions, collagen remains a key component of the extracellular matrix (ECM), which plays an essential role in regulating the wound-healing process. Chronic wounds are typically characterized by a hostile environment marked by persistent inflammation, excessive degradation of ECM components due to elevated levels of metalloproteinases (MMPs) and other enzymes, as well as being a consequence of the dysregulation of soluble mediators critical to wound-healing [46]. Data from an in vitro wound healing cell migration assay showed that the CK2 inhibitor CX-4945 attenuated MMP-1 secretion, thus inhibiting collagen degradation and cell migration, following downregulation of cyclic AMP response element-binding protein (CREB) and ERK mitogen-activated protein [47]. These data indicated that CK2 may regulate different pathways involved in collagen regulation, also depending on the conditions. In fact, Pei-Shan Wu et al. demonstrated that one of the mechanisms of cold atmospheric plasma jets (CAPJ) in promoting wound healing is also based on CK2 activation with the consequent induction of cell migration and ECM production during the late phase of wound healing. The results obtained in this study confirmed that keratinocyte migration is regulated by the bidirectional, phosphorylation-dependent activation of the PI3K/AKT and MAPK signaling pathways, orchestrated by CK2 kinase, that ultimately drive cell migration, intercellular communication, and ECM remodeling in wound healing [48]. The data described so far indicate that CK2 is a pivotal regulator in the pathogenesis of different skin diseases and influences key processes such as cell proliferation, differentiation, and inflammation. For this reason, CK2 targeting could offer a promising avenue for developing novel therapeutic strategies for skin disorders.

Ongoing research aims to further elucidate CK2’s role in skin diseases and to develop targeted therapies that can modulate its activity with high specificity and minimal side effects since poor data are still available.

## 4. CK2 Role in Skin Cancer

**Figure 3:**
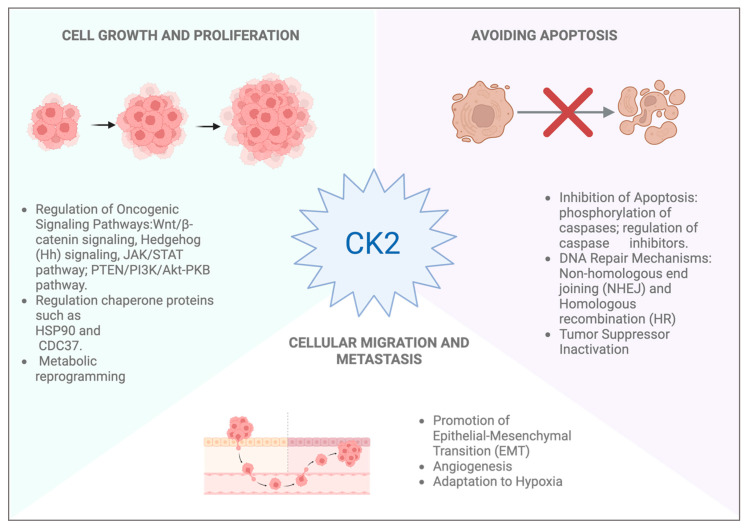
Representation of main hallmarks of CK2 in relation to cancer: CK2 promotes tumor progression by modulating key cancer-related processes. It contributes to cell growth and proliferation by regulating multiple oncogenic signaling pathways, including the Wnt/β-catenin, Hedgehog (HH), JAK/STAT and PI3K/Akt pathways. It also stabilizes chaperone proteins, such as HSP90 and CDC37, and influences metabolic reprogramming. Concurrently, CK2 supports cell survival by inhibiting apoptosis via the phosphorylation of caspases, the regulation of apoptosis inhibitors and the interference with DNA repair mechanisms such as non-homologous end joining (NHEJ) and homologous recombination (HR), as well as the inactivation of tumor suppressors. Furthermore, CK2 plays a pivotal role in cellular migration and metastasis by promoting epithelial-to-mesenchymal transition (EMT), enhancing angiogenesis, and enabling tumor adaptation to hypoxic conditions. Created in Biorender. Speranza. (2025) https://BioRender.com (accessed on 11 April 2025).

CK2 is frequently dysregulated in different types of cancer, including skin cancer, and its activity has been associated with several cancer hallmarks, such as uncontrolled cell proliferation, evasion of apoptosis and metastatic potential [49]. Among skin cancers, two main categories are recognized: cutaneous melanoma (CM) and non-melanoma skin cancers (NMSC), with the latter accounting for over 90% of all cases. NMSCs are further subdivided into basal cell carcinoma (BCC) and squamous cell carcinoma (SCC) that originate from epidermal keratinocytes. In contrast, CM is a malignant tumor originating from melanocytes and is classified into four major subtypes: superficial spreading melanoma (SSM), the most common subtype, lentigo malign melanoma (LMM), nodular melanoma (NM) and acral lentiginous melanoma (ALM) [50]. The pathogenesis of CM is multifactorial and incorporates both genetic and environmental influences. Several somatic mutations have been defined as important genetic factors; contributing mutations include V-Raf Murine Sarcoma Viral Oncogene Homolog B (*BRAF*) ~60%, Neuroblastoma RAS viral oncogene homolog (*NRAS*) ~20%, and PIK3 genes, as well as loss of neurofibromin 1 (NF1), deletions of cyclin-dependent kinase inhibitor 2A (CDKN2A), microphthalmia-associated transcription factor (MITF) amplifications ~10–20% and PTEN deletions [51]. About 25% of melanomas develop from benign nevi that have melanocytes with specific mutations, such as BRAFV600E or NRASQ61K, which are often associated with melanoma. However, in their benign state, these mutated melanocytes typically remain non-proliferative due to a phenomenon known as oncogene-induced senescence. This protective mechanism occurs when cells with mutations in oncogenes such as BRAFV600E, which is found in nearly 50% of melanoma patients, or NRASQ61K, undergo irreversible growth arrest. Nevertheless, the onset of further mutations coupled with additional changes in the environment may enable these senescent cells to escape this growth arrest and acquire malignant features, thereby progressing to melanoma [52]. Among the non-genetic factors, UV radiations show the most significant impact on melanoma progression. In fact, UV radiation exposure causes severe damage to nucleic acids, enhances mutagenesis, and inactivates the tumor suppressor protein p53, which is crucial for apoptosis induction as a response to cellular stress [53]. Both genetic and environmental factors work together in a troubling manner; they are involved in the development of and trigger melanoma. The association between CK2 and cancer has been well documented, with elevated kinase levels correlating with increased cell proliferation and resistance to apoptosis, thus contributing to tumor progression. As illustrated, CK2 regulates a number of important signaling pathways, which include the Wnt pathway by phosphorylating and stabilizing β-catenin, Hedgehog signaling, the JAK/STAT pathway, NF-κB, and the CK2-dependent PTEN/PI3K/Akt signaling pathway, where CK2 functionally abolishes the activity of the tumor suppressor PTEN [54]. Therefore, CK2 plays a pivotal role in cancer progression through different mechanisms that promote cell survival, proliferation and resistance to treatment (Figure 3).

CK2 further increases drug resistance by increasing the levels of P-glycoprotein efflux transporters, along with the multidrug resistance-associated protein 1 (MRP1) and the breast cancer resistance protein (BCRP) [4]. Additionally, CK2 inhibits apoptosis by phosphorylating caspase substrates, preventing pro-apoptotic BID’s activation and enhancing apoptosis inhibitors, such as the apoptosis repressor with caspase recruitment domain (ARC). As a result of these actions, tumor cells are capable of surviving chemotherapy, leading to increased treatment resistance [55]. CK2 also modulates oncogenic chaperones HSP90 and CDC37 and weakens the unfolded protein response (UPR) by inositol-requiring enzyme 1- X-box binding protein 1 (IRE1–XBP1) activation, as well as the protein kinase RNA-like endoplasmic reticulum kinase (PERK) mediated apoptosis. The balance shift towards tumor development is controlled by CK2, particularly in the destruction of tumor suppressors, p53, PTEN and Ikaros, which are removed through MDM2’s strengthening control. Changes in the metabolic profiles of cancer cells are also regulated by CK2, allowing cells to thrive and divide uncontrollably within harsh environments devoid of nutrients. Finally, CK2 enhances multiple hallmark traits of cancer, including cellular migration and invasion, epithelial-mesenchymal transition (EMT), tumor angiogenesis, and adaptation to hypoxia, which increase the aggressiveness of tumors by providing metastatic potential [4]. In melanoma, the prooncogenic activity of CK2 is mediated by the positive regulation of β-catenin stability, involved in both cell–cell adhesion and gene transcription. In this context, β-catenin plays a key role in the adherent junction that binds to the cytoplasmic domain of E-cadherin in order to anchor the cadherin complex to the cytoplasmic filamentous actin skeleton. This bond supports intracellular cohesion and maintains the structural integrity of epithelial tissue. In addition to its scaffolding function, β-catenin is a crucial member of Wnt signaling pathways and controls the transcription of different genes responsible for cell proliferation, tumor formation and other related activities. The binding of Wnt ligands to Frizzled (Fzd) and the LRP5/6 receptor activates disheveled (Dvl) signaling basic, resulting in the phosphorylation of Dvl cytoplasmic protein and activation of its role, leading to β-catenin destruction. Therefore, the cytoplasmic-accumulated β-catenin will also eventually move to the nucleus. In the nucleus, β-catenin combines with members of the TCF/LEF family transcription factors and with other cofactors like BCL9 and Pygopus, which results in their active participation in target gene expression, such as cell cycle regulatory genes, like cyclin D1 [56]. At several steps of this pathway, CK2 has a critical regulatory function by decreasing GSK3-mediated Dvl degradation, which enhances Dvl stabilization. Furthermore, it facilitates the transport of β-catenin to the nucleus and augments the transcriptional functions of β-catenin. In addition, CK2 also increases the activity of TCF/LEF transcription factors, which Wnt/β-catenin signaling responds to more [4]. Melanoma has a high correlation with the dysregulated activation of the Wnt/β-catenin pathway, which has been implicated in promoting both metastasis and invasion [56]. At the early stages of melanoma and in normal melanocytes, hyperactivation of Wnt signaling can induce EMT, thus raising the invasive capacity of the cells. Negative modulators of this pathway, such as RNF43, have been shown to limit invasion and increase the efficacy of selective agents [56]. Moreover, Dong et al. (2023) note that cell transition can also be driven by non-proliferative Fzd6 Wnt signaling related to cell invasiveness [57]. Besides that, studies have also highlighted the therapeutic potential of targeting Wnt/β-catenin signaling in melanoma. Wang et al. reported that the inhibition of this pathway sensitizes melanoma cells to ferroptosis and improves their response to anti-PD-1 immunotherapy [58]. Moreover, Wronski et al. demonstrated that receptor-interacting serine/threonine-protein kinase 4 (RIPK4) knockdown reduces LRP6 and β-catenin-mediated growth, migration and invasion of melanoma cells via Wnt3a activation, resulting in a significant decrease in tumor progression [59]. The functional role of β-catenin in the progression of melanoma has been further supported by studies undertaken using genetically engineered mouse models. Damsky et al. (2011) [60] showed that in the context of PTEN loss and BRAF activation, β-catenin functions as a key mediator of tumor progression and metastasis in melanoma. In these models, the genetic disabling of β-catenin resulted in delayed tumor onset, increased overall survival, and significantly lowered metastatic spread. On the other hand, metastatic spread to lymph nodes, lungs, the intestines, and the spleen (organs typically associated with advanced human melanoma) was accelerated by β-catenin stabilization, alongside reduced survival [60]. These findings highlight the pivotal role of endogenous β-catenin in melanoma origin, advancement, and spread.

Despite recent advances in therapy, melanoma remains one of the most aggressive and lethal forms of cancer. Current therapeutic strategies primarily focus on targeting oncogenic BRAF mutations by using specific inhibitors such as vemurafenib, dabrafenib, trametinib and immune checkpoint inhibitors in patients with high-risk or recurrent disease. Although these approaches have yielded clinical benefits, the development of resistance remains a major obstacle, reinforcing the need for alternative and complementary therapeutic approaches [61]. Because of its broad influence on key cellular mechanisms, CK2 has emerged as a compelling therapeutic target in melanoma. Zhou et al. demonstrated that CK2α plays a key role in conferring resistance to RAF-MEK-ERK pathway inhibitors in BRAF mutant melanoma. Although BRAF-targeted therapies demonstrate initial clinical efficacy, resistance frequently emerges over time. In fact, the study revealed that CK2α overexpression sustains ERK activity, even in the presence of BRAF and MEK inhibitors, by promoting melanoma progression and therapeutic resistance. Notably, this resistance is not dependent on CK2 kinase activity but rather on its scaffolding functions. CK2α was shown to interact with the kinase suppressor of Ras 1 (KSR1), a key scaffold protein required for efficient RAF-MEK-ERK signal transduction. Additionally, CK2α facilitates the degradation of the dual specificity phosphatase 6 (DUSP6), a phosphatase that specifically inactivates ERK, resulting in sustained ERK phosphorylation [62]. In this setting, the observation that a kinase-inactive CK2α mutant can still confer resistance further supports the importance of its protein-protein interaction capabilities over its enzymatic function. In order to support the involvement of CK2 in melanoma therapy resistance, Parker and colleagues investigated the effects of combining CK2 and BRAF inhibition. Melanoma cell lines with BRAF^V600E and BRAF^V600K mutations exhibited enhanced tumor cell killing and decreased cell viability when CX-4945, a selective CK2 inhibitor, was used with vemurafenib. The synergy measured with the combination index (CI) was 0.21, which is less than 1, indicating that the combination of BRAF and CK2 inhibition interacts more than additively, which is strong evidence for crosstalk between the two pathways. Paradoxically, these wild-type BRAF melanoma cells did not display this enhanced effect, indicating, perhaps, heightened CK2 dependency in cells with BRAF mutations. Melanoma cells also showed higher sensitivity to CK2 inhibition compared to other malignancies like thyroid cancer, exhibiting a 20–35% inhibition rate following CX-4945 treatment. This effect suggests that CK2 signaling has a heightened reliance on controlling signaling pathways in melanoma cells, making CK2 a strategic therapeutic target. Also, specific silencing of CK2α with siRNA in BRAF mutant cell line caused synergism with vemurafenib, which was not evident in wild-type cells [63]. These findings reinforce the functional relevance of CK2 in mediating resistance to BRAF inhibition and support the rationale for incorporating CK2-targeted approaches in therapeutic regimens aimed at improving clinical outcomes in patients with BRAF melanoma.

CK2 inhibitors have also been shown to be effective in basal cell carcinoma. Originating from totipotent hair follicle cells, BCC is generally characterized by low metastatic potential. Although most cases are successfully treated by surgical excision with low recurrence rates, approximately 1–10% may progress to an advanced stage, either as laBCC, for which surgery would result in disfigurement or functional impairment, or as mBCC, which carries a more unfavorable prognosis. In these forms, radiotherapy may be considered in selected unresectable cases, but systemic treatment remains the cornerstone for non-surgical or metastatic disease. The molecular pathogenesis of basal cell carcinoma (BCC) is primarily driven by the aberrant activation of the Hedgehog (HH) signaling pathway. In normal cells, the PTCH1 receptor suppresses Smo in the absence of HH ligands, thereby inhibiting the activation of downstream transcription factors. In BCC, when Shh ligands bind to PTCH1, this repression is lifted, and SMO activates glioma-associated oncogene (GLI) transcription factors. After dissociating from their inhibitor suppressor of fused (SUFU), these factors then translocate to the nucleus and drive the expression of genes involved in cell proliferation, survival and oncogenesis. CK2 is an important modulator of Hedgehog (HH) signaling, increasing the stability and transcriptional activity of GLI2. CK2 phosphorylates GLI2, thereby promoting its nuclear accumulation and protecting it from proteasomal degradation. This amplifies HH signaling independently of SMO [64]. This is clinically relevant because CK2 could be a therapeutic target for treating BCC, particularly in cases where tumors have developed resistance to SMO inhibitors such as vismodegib and sonidegib. Silmitasertib was tested in a Phase 1, multicenter, open-label clinical trial (NCT03897036) involving 25 patients with laBCC or mBCC who were previously SMO inhibitor therapy failures or were intolerant to therapy [65]. (Table 1).

Patients received 1000 mg orally twice a day in two 28-day cycles per cohort, starting with eight patients in the Treatment–Duration–Increment cohort, which concentrated on optimizing treatment duration, followed by seventeen patients in the Expansion cohort who assessed safety and efficacy at the set regimen. Of the 22 evaluable patients for efficacy, 3 (with laBCC) had a partial response, while 10 achieved disease stabilization. Disease control rates were calculated as 80% for metastatic BCC and 65% for locally advanced BCC. The median PFS was 9.2 months in patients with laBCC and 3.7 months in mBCC. Notably, two patients had PFS exceeding 21 months, suggesting the possibility of long-term disease control. Silmitasertib showed a tolerable safety profile. Treatment-emergent adverse events (TEAEs) occurred in 100% of subjects in the Treatment–Duration–Increment cohort and in 94.1% of subjects from the Expansion cohort, while serious adverse events (SAEs) were present in 25% and 17.7% of subjects, respectively. Among the more common AEs were diarrhea (100%; 82.4%), nausea (62.5%; 52.9%), and vomiting (37.5%; 23.5%). Other AEs included fatigue (25%; 11.8%) and asthenia (25%; 0%), followed by metabolism and nutrition disorders such as hypokalemia (12.5%; 23.5%) and hyperglycemia (12.5%; 11.8%). Infectious events such as oral candidiasis and pneumonia occurred more frequently in the larger Expansion cohort. It is noteworthy that the treatment discontinuation rate due to adverse events was 24%, which is considered favorable compared to currently approved HH inhibitors. The study results suggest that targeting CK2 with silmitasertib could be clinically beneficial for patients with advanced BCC, particularly those who are resistant to Smo inhibition.

## 5. Conclusions

Among the various pharmacological inhibitors developed to target CK2, CX-4945 is the most extensively studied compound. It is a selective ATP-competitive inhibitor of CK2 that showed potent activity in preclinical models of cancer and inflammatory diseases. Other inhibitors, such as CX-5011 and chemical probes, like GO289, have demonstrated efficacy in experimental settings by inhibiting CK2 activity and reducing disease-associated phenotypes [66]. CK2 inhibitors have entered clinical trials, and, in this context, CX-4945 showed promising results in Phase I and II studies for different cancer treatments [67]. Although these trials primarily focus on oncology, the anti-inflammatory properties of CK2 inhibitors highlight their potential use in dermatological applications, such as psoriasis and atopic dermatitis [5,68]. However, challenges remain, including potential off-target effects and the need for improved delivery mechanisms to ensure localized action in the skin. In fact, achieving effective and targeted delivery of CK2 inhibitors to the skin remains a technical hurdle. The skin barrier function, primarily governed by the stratum corneum, poses a significant obstacle to the penetration of topically applied drugs. Strategies such as nanoparticle-based delivery systems, prodrugs, or chemical enhancers are being explored to overcome this limitation, but their clinical translation is still in the early stages. Therefore, achieving localized, controlled, and sustained release of CK2 inhibitors specifically to diseased or wounded skin remains a critical area for future research.

The development of clinically viable CK2 inhibitors has also been hindered by issues related to specificity, bioavailability, and pharmacokinetics. One major limitation is the ubiquitous expression and constitutive activity of CK2 in normal cells, which raises concerns about off-target effects and toxicity. In fact, CK2 regulates multiple signaling pathways and cellular functions, making selective inhibition difficult without disrupting normal physiological processes. Even partial inhibition can induce cytotoxicity in non-target tissues, potentially causing adverse effects that limit the therapeutic window. Clinical studies have reported dose-limiting toxicities with some CK2 inhibitors, emphasizing the need for a more refined approach to minimize systemic exposure. Moreover, resistance mechanisms and compensatory pathway activation may reduce the long-term efficacy of CK2 inhibitors.

In this context, the administration of combined therapies that include CK2 inhibitors and other targeted agents may offer synergistic benefits for managing complex skin disorders. Future research on CK2 should focus on its unexplored roles in skin physiology and disease; moreover, understanding CK2 interactions with other signaling molecules may uncover novel pathways that influence skin diseases. Advanced technologies, such as CRISPR-based screens and single-cell analysis, could provide deeper insights into CK2-specific roles in skin cells by considering different cell populations.

## Figures and Tables

**Table 1:** Silmitasertib (CX-4945) clinical study in advanced BCC.

Study Type	Phase I, multicenter, open-label clinical trial
Clinical Trial ID	NCT03897036
Patient Population	A total of 25 patients with locally advanced BCC (laBCC) or metastatic BCC (mBCC) who had prior failure or intolerance to Smo inhibitors
Dosing Regimen	A total of 1000 mg orally twice daily, administered over two 28-day cycles
Initial Cohort	A total of 8 patients in the Treatment–Duration–Increment cohort (focused on optimizing treatment duration)
Expansion Cohort	A total of 17 patients (evaluated safety and efficacy at the established dosing regimen)
Efficacy	Among 22 evaluable patients: 3 (with laBCC) achieved partial response, 10 had stable disease
Disease Control Rate (DCR)	A total of 80% in mBCC, 65% in laBCC
Median Progression-Free Survival (PFS)	A total of 9.2 months (laBCC), 3.7 months (mBCC)
Long-term Response	A total of 2 patients experienced PFS > 21 months

## Abbreviations

The following abbreviations are used in this manuscript:

AKT	Ak strain transforming/Protein kinase B
ALM	Acral lentiginous melanoma
ATP	Adenosine Triphosphate
α-TAT1	α-tubulin acetyltransferase
AP-1	Activator protein 1
ARC	Apoptosis repressor with caspase recruitment domain
BCC	Basal cell carcinoma
BCL9	B-cell lymphoma 9 protein
Bcl-xL	B-cell lymphoma-extra large
BCRP	Breast cancer resistance protein
BRAF	V-Raf Murine Sarcoma Viral Oncogene Homolog B
CAPj	Cold atmospheric plasma jet
CDKN2A	Cyclin-dependent kinase inhibitor 2A
Ci	Cubitus interruptus
CM	Cutaneous melanoma
CK2	Casein kinase 2
CX-4945	Silmitasertib
CREB	cyclic AMP response element-binding protein
dcSSc	Diffuse cutaneous systemic sclerosis
DUSP6	Dual specificity phosphatase 6
Dvl	Dishevelled
ECM	Extracellular Matrix
ERK	Extracellular signal-regulated kinase
EMT	Epithelial-mesenchymal transition
Fzd	Frizzled receptor
GSK3	Glycogen Synthase Kinase 3
IKB-α	NF-kappa-B inhibitor alpha
IKKγ	IκB kinase-γ
IFN-γ	Interferon Gamma
IRE1	Inositol-requiring enzyme 1
JAK	Janus kinase
KSR1	Kinase suppressor of Ras 1
LEF	Lymphoid enhancer-binding factor
lcSSc	limited cutaneous systemic sclerosis
LMM	Lentigo maligna melanoma
LRP5/6	Low-density lipoprotein receptor-related protein 5/6
MAPK	Mitogen-activated protein kinase
MITF	Microphthalmia-associated transcription factor
MMPs	Matalloproteinases
MRP1	Multidrug resistance-associated protein 1
NEMO	NF-κB essential modifier
NF1	Neurofibromin 1
NM	Nodular melanoma
NMSC	Non-melanoma skin cancers
NF-kB	Nuclear Factor-kappa B
NRAS	Neuroblastoma RAS viral oncogene homolog
PDK1	Phosphoinositide-Dependent Kinase-1
P-gp	P-glycoprotein
PERK	Protein kinase RNA-like endoplasmic reticulum kinase
PI3K	Phosphoinositide 3-kinase
PTEN	Phosphatase and tensin homolog
RIPK4	Receptor-interacting serine/threonine-protein kinase 4
RNF43	Ring Finger Protein 43
SCC	Squamous cell carcinoma
SMAD	Suppressor of Mothers Against Decapentaplegic
SMO	GPCR-like protein Smoothened
SSc	Systemic Sclerosis
SSM	Superficial spreading melanoma
SOCS3	Suppressor of Cytokine Signaling 3
STAT	Signal transducer and activator of transcription
TBB	4,5,6,7-tetrabromobenzotriazole
TCF	T cell factor
TGF-ß	Transforming Growth Factor-beta
UPR	Unfolded protein response
UV	Ultraviolet
Wnt	Wingless/Integrated
XBP1	X-box binding protein 1
